# Synergistic Flame Retardancy of Epoxy Resin with Aminated Multi-Walled Carbon Nanotubes and Ammonium Polyphosphate

**DOI:** 10.3390/polym18101158

**Published:** 2026-05-08

**Authors:** Yiwen Wang, Qian Hu, Miaojia Ye, Xiaoyue Huang, Quankai Chen, Chuanqun Hu

**Affiliations:** School of Materials and Chemical Engineering, Hubei University of Technology, Wuhan 430068, China; 13367232750@163.com (Y.W.); 15827225228@163.com (Q.H.); 15623677316@163.com (M.Y.); huangxiaoyue0204@163.com (X.H.); m13177330069@163.com (Q.C.)

**Keywords:** epoxy resin, flame retardant, multi-walled carbon nanotubes, ammonium polyphosphate

## Abstract

Epoxy resins have been extensively applied in aerospace and automotive fields. Nevertheless, their inherent flammability significantly restricts broader applications. In this study, carboxylated multi-walled carbon nanotubes (COOH-MWCNTs) were first aminated to obtain aminated Multi-Walled Carbon Nanotubes (NH_2_-MWCNTs). Subsequently, NH_2_-MWCNTs and ammonium polyphosphate (APP) were incorporated into the epoxy resin via mechanical stirring, thereby constructing a phosphorus–carbon synergistic flame-retardant system. Compared with the neat epoxy thermoset, the EP/17.5APP/0.1NH_2_-MWCNTs composite showed a limiting oxygen index (LOI) value of 29.6% and attained a UL-94 V-0 rating. In addition, for the modified composite material, the maximum thermal decomposition rate (R_Tmax_) is 12.4 wt%/min, the char residue at 600 °C (C_600_) reaches 44.2%, and the smoke density is 425.8. The impact strength and tensile modulus are increased to 10.1 Mpa and 3.0 Gpa, respectively, while the compressive strength remains essentially unchanged. Furthermore, the synergistic flame-retardant mechanism between phosphorus and carbon was investigated by analyzing the char residues of the epoxy resin and its composites. This study offers a promising approach for designing epoxy composites with improved flame retardancy and enhanced thermal stability for high fire-safety applications, such as electronic encapsulation and structural materials.

## 1. Introduction

Epoxy resin (EP), characterized by excellent adhesion, corrosion resistance, high hardness, and superior mechanical properties, has been extensively applied in mechanical manufacturing, aerospace, electronics, and chemical anti-corrosion fields [1]. However, the inherent high flammability of epoxy resin severely restricts its application potential, highlighting the necessity of flame-retardant modification. Driven by increasing concerns over environmental safety and human health, halogen-free flame retardants have gradually become the preferred alternative to halogen-containing systems in epoxy resin flame-retardant applications [2,3].

In recent years, phosphorus-based flame retardants have received increasing attention in the field of flame-retardant materials and are progressively regarded as an important alternative to conventional halogenated flame retardants [4]. Compared with conventional halogen-containing systems, phosphorus-based flame retardants mainly exert their effects by promoting the formation of a dense char layer during combustion and inhibiting the transfer of free radicals in the flame, thereby exhibiting the advantages of low loading and high flame-retardant efficiency. More importantly, they generate fewer toxic fumes and corrosive gases during thermal decomposition and combustion, significantly reducing fire hazards and better meeting the current requirements for environmental friendliness and fire safety of materials. Owing to these characteristics, phosphorus-containing compounds are widely recognized as environmentally friendly and highly efficient flame retardants, showing broad application prospects in flame-retardant polymer materials for electronics, construction, and transportation fields [5]. Ammonium polyphosphate (APP), an inorganic phosphorus-based flame retardant containing both phosphorus and nitrogen elements, has been widely used in epoxy resin (EP) systems due to its low cost, low toxicity, and high flame-retardant efficiency [6,7]. However, the use of APP alone suffers from several drawbacks, including high loading requirements, poor compatibility with epoxy resin, and deterioration of the mechanical properties of the composites. Therefore, it is necessary to introduce synergistic flame retardants in combination with APP to further enhance the flame-retardant performance of epoxy resin [8].

With the rapid development of preparation techniques and performance optimization of inorganic nanomaterials, nano-reinforced polymer composites have become one of the most promising research directions in the field of materials science. In this area, carbon nanotubes (CNTs) have attracted extensive attention from both academia and industry due to their unique structural characteristics and outstanding physicochemical properties [9]. Meanwhile, the application of CNTs in the field of flame-retardant materials has also gained increasing interest. During combustion, the incorporation of CNTs can facilitate the formation of a protective carbon layer, resulting in a denser and more robust char residue, which can effectively block heat and mass transfer between the polymer matrix and the surrounding environment [9,10]. Yu et al. [11] grafted molybdenum-phenolic resin (Mo-PR) onto the surface of MWCNTs, which improved the dispersion of MWCNTs in epoxy resin. The CNT-PR was then compounded with melamine and blended with epoxy resin to prepare epoxy composites, whose flame-retardant performance was subsequently evaluated. The results showed that with the addition of 5 wt% CNT-PR and 8 wt% melamine, the composites achieved a UL-94 V-0 rating. Shi et al. [12] modified MWCNTs using the silane coupling agent KH-570 and melamine phosphate (MPP) to obtain the flame-retardant CNTs-M. Compared with pristine MWCNTs, CNTs-M exhibited improved compatibility with epoxy resin. The CNTs-M was incorporated into epoxy resin to prepare composites, whose flame-retardant performance was then evaluated. The results showed that with 10 wt% CNTs-M, the composites achieved a limiting oxygen index (LOI) of 28.3% and a UL-94 vertical burning rating of V-2. In addition, the interfacial thermal resistance between the modified MWCNTs and epoxy resin was reduced, resulting in enhanced thermal conductivity of the composites. This functional modification strategy provides a promising approach for designing epoxy composites with both improved flame retardancy and thermal conductivity.

Despite considerable progress in APP-based and carbon nanomaterial-assisted flame-retardant epoxy systems, several challenges remain, including the high loading requirement of APP, poor interfacial compatibility, and the agglomeration tendency of carbon nanofillers, which limit their synergistic efficiency and often compromise mechanical properties. Although functionalized carbon nanotubes have been explored, achieving effective flame retardancy at ultralow nanofiller loading while maintaining structural integrity remains challenging.

To address the inherent flammability of epoxy resin, COOH-MWCNTs were first aminated to obtain NH_2_-MWCNTs. Amino functionalization introduces reactive sites that can participate in epoxy curing reactions, thereby enhancing interfacial interactions and improving the dispersion of nanofillers within the epoxy matrix [13]. Meanwhile, ammonium polyphosphate (APP), although widely recognized as an efficient phosphorus–nitrogen flame retardant, is inherently hygroscopic and generally requires relatively high loading, which may adversely affect its compatibility and long-term application stability in epoxy systems. In view of these limitations, this study further combines NH_2_-MWCNTs with APP at an optimized ratio to construct a synergistic flame-retardant system for epoxy resin composites through mechanical stirring and a thermal curing process.

Compared with conventional APP-filled epoxy systems, this work shows that the addition of a low content of amino-functionalized multi-walled carbon nanotubes further enhances the flame-retardant efficiency of APP, while simultaneously improving char compactness and structural integrity. More importantly, amino functionalization strengthens the interfacial interaction between carbon nanotubes and the epoxy matrix, leading to improved nanofiller dispersion and a more effective condensed-phase synergistic effect with APP. This phosphorus–carbon–nitrogen synergistic strategy provides a promising approach for improving the fire safety of epoxy composites while largely preserving their main mechanical performance.

## 2. Materials and Methods

### 2.1. Raw Materials

Epoxy resin (EP) (E-51) was purchased from Shandong Uso Chemical Technology Co., Ltd. (Linyi, China); Carboxylated multi-walled carbon nanotubes (COOH-MWCNTs) (>95%) were purchased from Shanghai Macklin Biochemical Co., Ltd. (Shanghai, China); N,N-Dimethylformamide (DMF) (AR) was purchased from Shanghai Aladdin Biochemical Technology Co., Ltd. (Shanghai, China); Ethylenediamine (EDA) (AR) was purchased from Sinopharm Group Chemical Reagent Co., Ltd. (Shanghai, China); Tetramethyluronium hexafluorophosphate (HATU) (98%) was purchased from Shanghai Macklin Biochemical Co., Ltd. (Shanghai, China); 4,4′-diaminodiphenylmethane (DDM) (98.0%) was purchased from Guangzhou Jinrui Chemical Co., Ltd. (Guangzhou, China); Ammonium polyphosphate (APP) (n ≥ 1000) was purchased from Shanghai Macklin Biochemical Co., Ltd. (Shanghai, China).

### 2.2. Modification of Carboxylated Multi-Walled Carbon Nanotubes

**Figure 1:**
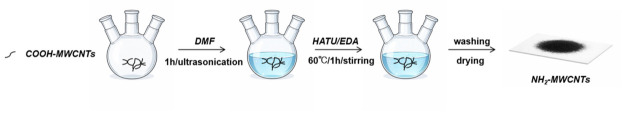
Preparation pathway of NH_2_-MWCNTs.

COOH-MWCNTs were initially dispersed in DMF and ultrasonicated at 30 °C for 1 h. Then, ethylenediamine (EDA) was introduced, and the mixture was subjected to further ultrasonication for another 1 h. After sonication, HATU was added to the mixture, which was subsequently transferred into a 500 mL three-necked round-bottom flask. The reaction was conducted at 60 °C under magnetic stirring and reflux conditions for 6 h to obtain crude NH_2_-MWCNTs. The resulting product was then diluted, washed, and filtered with ethanol; this purification process was repeated three times to yield purified NH_2_-MWCNTs. Finally, the obtained NH_2_-MWCNTs were vacuum-dried at 50 °C for 24 h for further use. The modification procedure of MWCNTs is illustrated in Figure 1.

### 2.3. Preparation of EP/APP/NH_2_-MWCNTs Composite Materials

**Figure 2:**
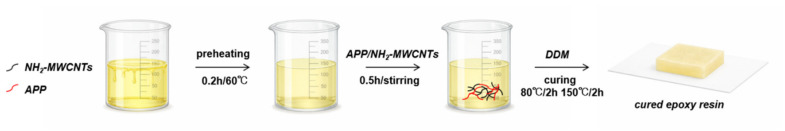
Preparation process of cured epoxy resin.

The epoxy resin was first preheated to 60 °C to improve its processability. Subsequently, APP and NH_2_-MWCNTs were incorporated into the resin and mechanically stirred at this temperature for 30 min to ensure uniform dispersion. DDM was then added as the curing agent, followed by continuous stirring for 20 min. Finally, the mixture was poured into a polytetrafluoroethylene mold, cured at 80 °C for 2 h, and post-cured at 150 °C for 2 h to ensure complete crosslinking. The preparation process of the cured epoxy resin is shown in Figure 2. The formulation is presented in Table 1.

### 2.4. Measurement and Characterisation

#### 2.4.1. Infrared Characterization of Modified Multi-Walled Carbon Nanotubes

A small amount of the dried sample was thoroughly ground with potassium bromide (KBr) at a mass ratio of 1:150 using an agate mortar. The obtained mixture was then analyzed by Fourier transform infrared spectroscopy (FT-IR) in the wavenumber range of 500–4000 cm^−1^.

The dried multi-walled carbon nanotubes before and after modification were characterized by X-ray diffraction (XRD). The scanning range was set from 5° to 90°, with a scanning rate of 10°/min.

The dried multi-walled carbon nanotube samples before and after modification were uniformly dispersed on glass slides and analyzed using a confocal Raman spectrometer (XploRA Plus) (Palaiseau, France). The excitation wavelength was 532 nm.

X-ray photoelectron spectroscopy (XPS) analysis was performed using a PHI 5000 Versaprobe I (Chigasaki, Japan) system to determine the elemental composition and chemical states of the samples.

The pristine and modified multi-walled carbon nanotube samples were first dried and subsequently sputter-coated with gold to enhance their surface conductivity. The microstructural morphology of the samples was then observed using an SU-8010 (Tokyo, Japan) field-emission scanning electron microscope at an accelerating voltage of 10 kV.

#### 2.4.2. Performance Testing of Epoxy Resin and Its Composites

The limiting oxygen index (LOI) of the epoxy resin and its composites was measured in accordance with GB/T 2406.2-2009. The specimens were prepared with dimensions of 120.0 mm × 10.0 mm × 3.5 mm. The top-surface ignition method was employed, in which the innermost part of the flame was applied to the top surface of the specimen for 30 s, with the flame removed once every 5 s during this period. If the ignition time exceeded 180 s or the burning length exceeded 50 mm, the corresponding oxygen concentration was considered insufficient to sustain self-extinguishing, and the oxygen concentration was reduced before repeating the procedure. Each sample was tested at least five times to ensure the accuracy and reliability of the data.

The UL-94 vertical burning classification of the epoxy resin and its composites was evaluated in accordance with GB/T 2408–2021. The specimens were prepared with dimensions of 120.0 mm × 10.0 mm × 3.5 mm. Ignition was applied at a position 10 mm below the center of the specimen using a burner oriented at an angle of 45° to the horizontal. The samples were ignited twice, with each ignition lasting 10 s. The combustion behavior and the occurrence of dripping were recorded. Each group of samples was tested at least five times to ensure the accuracy and reliability of the data.

Smoke density tests were conducted on epoxy resin and its composite samples in accordance with ISO 5659-2 standard. The test mode was non-flaming combustion, with a radiant heat flux of 50 kW/m^2^. The sample dimensions were 75 mm × 75 mm × 25 mm.

Thermal stability of the epoxy resin and its composites was evaluated using a thermogravimetric analyzer (TGA). Prior to testing, the samples were dried to remove residual moisture. Approximately 5 mg of each sample was placed in an alumina crucible and heated from room temperature to 600 °C at a heating rate of 10 °C/min under a nitrogen atmosphere.

The tensile and compressive properties of the epoxy resin and its composites were evaluated in accordance with GB/T 2567–2021 using a WDW-100 electronic universal testing machine (Shanghai, China). The crosshead speed was set at 10 mm/min for tensile testing and 5 mm/min for compression testing. At least three parallel tests were conducted for each group of specimens, and the final results were reported as the average values.

#### 2.4.3. Char Residue Analysis of Epoxy Resin and Its Composites

The microstructural morphology of the dried char residues was observed using an SU-8010 field-emission scanning electron microscope at an accelerating voltage of 10 kV, in order to elucidate the flame-retardant mechanism of the additives.

To evaluate the degree of graphitization, the dried char residues were ground into powder and uniformly dispersed on a glass slide, followed by analysis using an XploRA Plus confocal Raman spectrometer. The measurements were carried out with a laser wavelength of 532 nm to characterize the structural ordering of the char residues.

X-ray photoelectron spectroscopy (XPS) was performed on the char residues using a PHI 5000 VersaProbe I system to determine the surface elemental composition and chemical states of the samples.

## 3. Results and Discussion

### 3.1. Amination Modification of COOH-MWCNTs

**Figure 3:**
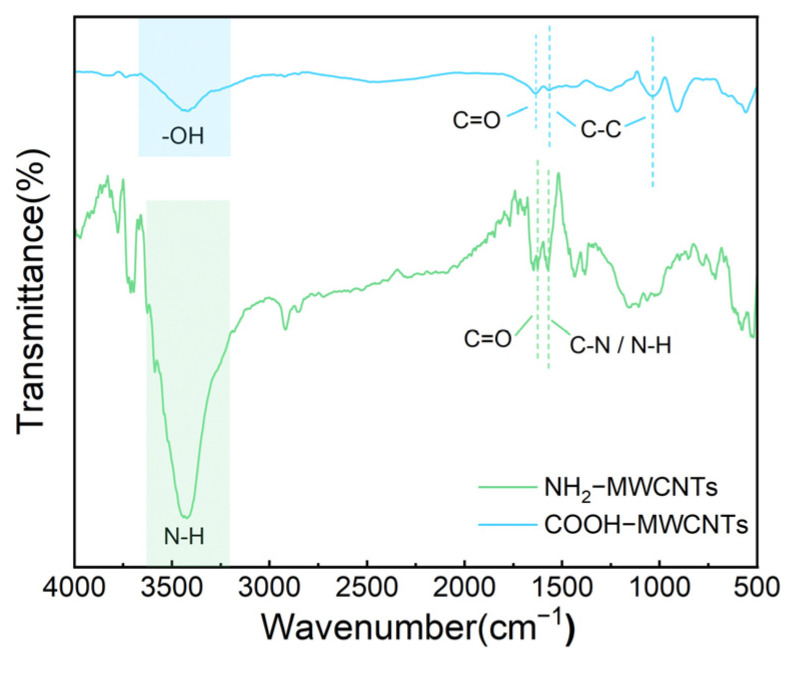
FT-IR spectra of COOH-MWCNTs and NH_2_-MWCNTs.

The FT-IR spectra of COOH-MWCNTs and NH_2_-MWCNTs are shown in Figure 3. For COOH-MWCNTs, the broad absorption band around 3400 cm^−1^ is attributed to the O-H stretching vibration, while the peak near 1700 cm^−1^ corresponds to the C=O stretching vibration. After modification, NH_2_-MWCNTs exhibit an enhanced absorption band at approximately 3430 cm^−1^, which is associated with the N–H stretching vibration. In addition, the peak at 1580 cm^−1^ can be attributed to the combined contributions of C-N stretching and N-H bending vibrations, while the band around 1620 cm^−1^ is assigned to the C=O stretching of amide groups. These results indicate that ethylenediamine has been successfully grafted onto the COOH-MWCNTs.

**Figure 4:**
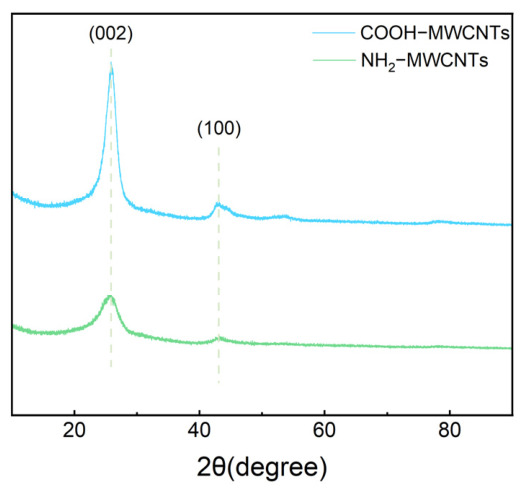
XRD patterns of COOH-MWCNTs and NH_2_-MWCNTs.

Figure 4 presents the X-ray diffraction (XRD) patterns of COOH-MWCNTs before and after modification. As observed, distinct diffraction peaks appear at 2θ = 26° and 44° for both samples, corresponding to the (002) and (100) planes of carbon materials, respectively. A comparison of the patterns before and after amination reveals that there are no significant shifts in peak position or noticeable changes in peak shape and width. This indicates that the modification with ethylenediamine occurs primarily on the surface of the carbon nanotubes and does not significantly affect their crystalline structure.

**Figure 5:**
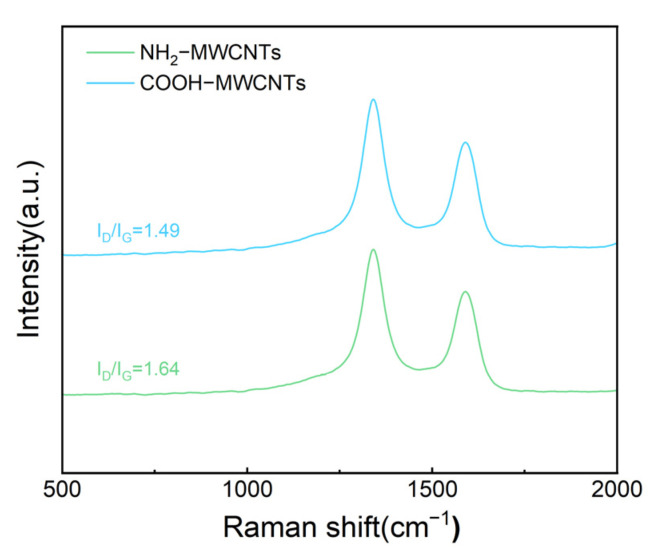
Raman spectra of COOH-MWCNTs and NH_2_-MWCNTs.

Figure 5 shows the Raman spectra of COOH-MWCNTs before and after modification. Prominent characteristic peaks are observed at 1342 cm^−1^ and 1590 cm^−1^, where the peak at 1342 cm^−1^ (D band) corresponds to sp^3^-hybridized carbon atoms, and the peak at 1590 cm^−1^ (G band) corresponds to sp^2^-hybridized carbon atoms. The intensity ratio of the D band to the G band (I_D_/I_G_) is commonly used to characterize the degree of structural disorder in carbon materials. The I_D_/I_G_ value of COOH-MWCNTs is approximately 1.49, while it increases to 1.64 after amination. This result indicates that the originally ordered sp^2^-hybridized structure of the MWCNTs is partially disrupted during the functionalization reaction with ethylenediamine, leading to an increase in sp^3^-hybridized content. The EDA molecules are thus successfully grafted onto the surface of COOH-MWCNTs via amide formation.

**Figure 6:**
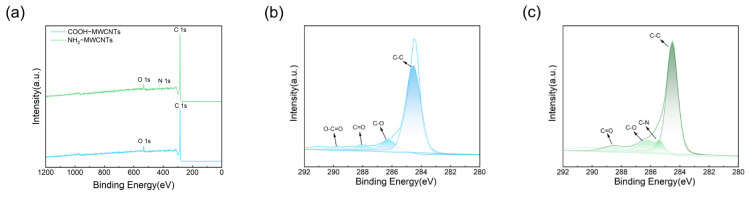
(**a**) XPS survey spectra of COOH-MWCNTs and NH_2_-MWCNTs; (**b**) C 1s spectrum of COOH-MWCNTs; (**c**) C 1s spectrum of NH_2_-MWCNTs.

X-ray photoelectron spectroscopy (XPS) was used to analyze COOH-MWCNTs before and after modification. As shown in Figure 6, the survey spectrum of COOH-MWCNTs exhibits two prominent peaks at 285.3 eV and 532.4 eV, corresponding to C 1s and O 1s, respectively, with no detectable N 1s signal, indicating that the surface of the material is primarily composed of carbon and oxygen [14,15]. After modification with ethylenediamine (EDA), a new peak appears in the 399–401 eV range in addition to the C 1s and O 1s signals, corresponding to N 1s. Detailed analysis of the C 1s high-resolution spectrum shows that COOH-MWCNTs display peaks at 284.5 eV (C-C), 286.3 eV (C-O), 288.1 eV (C=O), and 289.8 eV (O-C=O). Compared with COOH-MWCNTs, the modified material shows a new peak at 285.4 eV in the C 1s spectrum, assigned to the C–N bond [16]. This directly confirms the successful grafting of amino groups onto the carbon nanotube surface through EDA modification.

**Figure 7:**
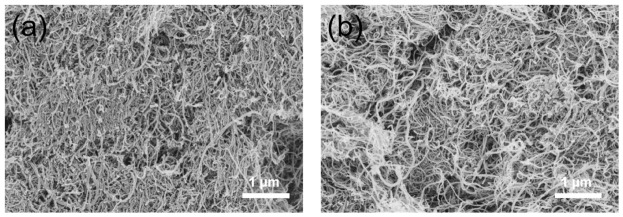
SEM images of (**a**) COOH-MWCNTs and (**b**) NH_2_-MWCNTs.

Figure 7 presents the SEM images of COOH-MWCNTs before and after modification. As shown, the pristine COOH-MWCNTs exhibit a relatively dense stacked structure with noticeable agglomeration between the nanotubes. This phenomenon is primarily attributed to the strong intermolecular hydrogen bonding among the surface carboxyl groups, which enhances the interactions between the nanotubes. After modification with EDA, the resulting material shows a significantly reduced stacking density, with an overall more loosely packed morphology. This indicates that the introduction of amino functional groups partially weakens the original hydrogen bonding between COOH-MWCNTs, reducing inter-tube interactions and thereby inhibiting severe agglomeration [17]. Such a relatively loose structure may be beneficial for improving the dispersion tendency in epoxy resin, as reduced inter-tube interactions can potentially suppress agglomeration during processing.

### 3.2. Flame-Retardant Performance of Epoxy Resin and Its Composites

In the evaluation of flame-retardant performance, limiting oxygen index (LOI) testing and UL-94 classification are two commonly used and representative methods. Table 2 summarizes the LOI values and UL-94 results for epoxy resin (EP) composites containing varying amounts of APP and NH_2_-MWCNTs. As shown in the table, the neat cured epoxy resin exhibits an LOI of only 21.5% and fails the UL-94 test, with significant dripping observed during combustion, indicating a high flammability. The introduction of APP alone significantly improved the flame retardancy of epoxy resin. Specifically, the EP/17.5APP composite exhibited an LOI value of 28.6% and achieved a UL-94 V-0 rating. This improvement can be attributed to the decomposition of APP during combustion, which produces phosphoric acid and polyphosphoric acid that promote rapid degradation and charring of the resin matrix [18]. The decomposition products of APP are also highly viscous, forming a dense insulating char layer that prevents oxygen from penetrating the matrix and inhibits the release of combustible gases, thereby hindering sustained burning. After the further introduction of MWCNTs on the basis of APP, the flame-retardant performance of the composites was further improved. The EP/17.5APP/0.1NH_2_-MWCNTs composite exhibited an LOI value of 29.6%, while the EP/17.5APP/0.1COOH-MWCNTs composite showed an LOI of 29.0%. Both composites achieved a UL-94 V-0 rating. This is attributed to the formation of a uniform carbonaceous layer derived from MWCNTs during combustion, which acts as a protective barrier, reducing ignition sensitivity and limiting the contact between the material and atmospheric oxygen [19]. Notably, due to the better dispersion and interfacial interaction of NH_2_-MWCNTs compared with COOH-MWCNTs in the epoxy matrix, the flame-retardant performance of EP/17.5APP/0.1NH_2_-MWCNTs is superior to that of EP/17.5APP/0.1COOH-MWCNTs. Overall, the APP/NH_2_-MWCNTs flame-retardant system effectively improves the flame-retardant performance of epoxy resin composites.

### 3.3. Smoke Density Testing of Epoxy Resin and Its Composites

**Figure 8:**
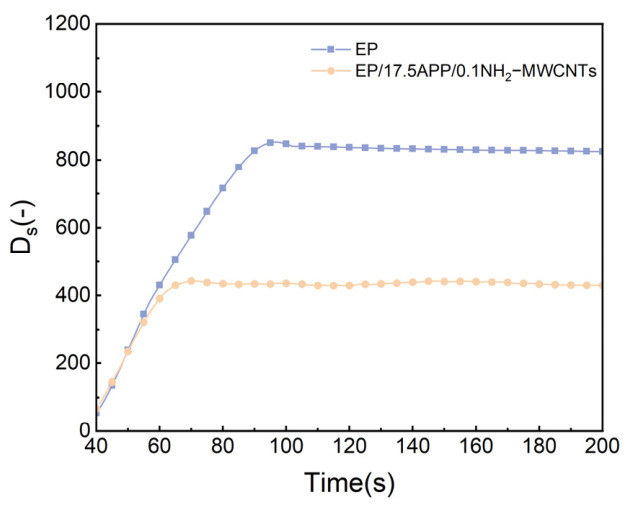
Smoke Density Curve of Epoxy Resin and Its Composites.

The smoke density curves of epoxy resin and its composites are presented in Figure 8. As shown, the neat epoxy exhibits a rapid increase in specific optical density (D_s_), reaching a high D_s,max_ of 820.7, indicating severe smoke generation during thermal decomposition. In contrast, the EP/17.5APP/0.1NH_2_-MWCNTs composite shows a markedly reduced D_s,max_ of 425.8, corresponding to an approximately 48% reduction. In addition, the modified composite reaches the steady-state stage earlier, suggesting that the combustion process is effectively suppressed. The improved smoke suppression behavior can be attributed to the synergistic action of APP and NH_2_-MWCNTs. During heating, APP decomposes to form phosphoric acid–based species along with non-flammable gases, which facilitate the formation of a continuous intumescent char layer. Meanwhile, NH_2_-MWCNTs further reinforce and densify the char structure, enhancing its barrier effect against heat transfer as well as the diffusion of combustible volatiles and smoke particulates. Consequently, the release of pyrolysis products is significantly hindered.

### 3.4. Thermal Stability of Epoxy Resin and Its Composites

**Figure 9:**
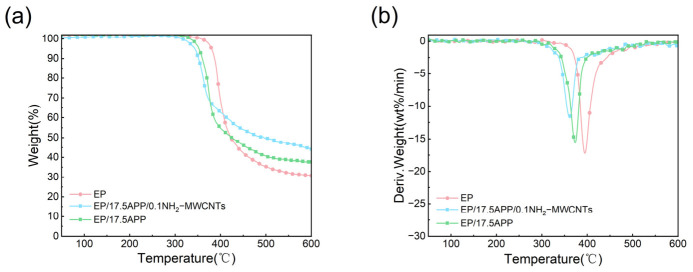
TG curves of EP and its composites in N_2_ (**a**) and DTG curves (**b**).

The thermal stability of epoxy resin and its composites was investigated by thermogravimetric analysis (TGA), with the TG and DTG curves presented in Figure 9 and the corresponding thermal data listed in Table 3. As shown, the neat cured epoxy resin exhibits a significant mass loss between 300–500 °C under a nitrogen atmosphere, which is primarily attributed to the breakdown of the epoxy network structure [20]. The T_5%_ (5 wt% mass loss temperature) and T_max_ (maximum decomposition rate temperature) of the neat epoxy resin are 381.5 °C and 395.9 °C, respectively, with only 31.1 wt% char remaining at 600 °C, indicating relatively limited thermal stability and charring ability. Upon the incorporation of flame retardants, the thermal decomposition behavior of the epoxy composites changes noticeably. After modification, both the T_5%_ and T_max_ of the composites decrease. Specifically, the T_5%_ of the EP/17.5APP and EP/17.5APP/0.1NH_2_-MWCNTs composites decreases to 350.8 °C and 333.4 °C, respectively, while their T_max_ decreases to 373.5 °C and 359.7 °C, respectively. The earlier T_5%_ is attributed to the polyphosphoric acid generated from APP decomposition, which catalyzes the thermal degradation and cross-linking of the epoxy matrix [21]. Compared with neat cured epoxy resin, the modified composites exhibited increased char residue at 600 °C (C_600_). In particular, the EP/17.5APP/0.1NH_2_-MWCNTs composite showed a more pronounced increase in char yield, reaching 44.2 wt%. This enhancement is attributed not only to the catalytic effect of APP in promoting dehydration and char formation at elevated temperatures, but also to the reinforcing effect of NH_2_-MWCNTs in the condensed phase, which facilitates the formation of a more compact and thermally stable protective char layer [22,23]. From the DTG curves, it can be further observed that the maximum mass loss rate of EP/17.5APP/0.1NH_2_-MWCNTs is significantly lower than that of the neat epoxy resin, indicating a slower decomposition process after modification. This suggests that the char layer formed during combustion can partially block heat transfer and inhibit the release of combustible gases, thereby reducing the thermal degradation rate of the material.

In summary, although the incorporation of APP lowers the initial thermal decomposition temperature of the composites, it significantly enhances their char-forming ability. The further introduction of NH_2_-MWCNTs promotes the formation of a more compact and stable char layer, thereby improving the high-temperature thermo-oxidative stability and flame-retardant performance of the composites.

### 3.5. Mechanical Properties Testing of Epoxy Resin and Its Composites

**Figure 10:**
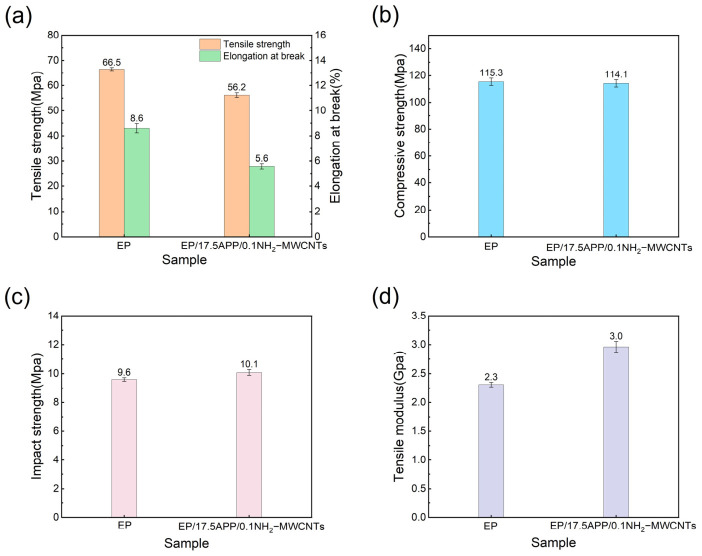
Mechanical properties of EP and its composites: (**a**) tensile strength; (**b**) compressive strength; (**c**) impact strength; (**d**) tensile modulus.

The mechanical property test results of epoxy resin and its composites are shown in Figure 10. As shown in Figure 10a, the tensile strength and elongation at break of the neat epoxy resin are 66.5 MPa and 8.6%, respectively, while those of the modified composite decrease to 56.2 MPa and 5.6%. Compared with neat epoxy resin, both tensile strength and elongation at break of the flame-retardant composite are reduced. This reduction can be attributed to the relatively high loading of APP, which tends to introduce stress concentration sites at the interface and facilitates crack initiation and propagation under tensile loading. As shown in Figure 10b, the compressive strength of the neat epoxy resin is 115.35 MPa, while that of the flame-retardant composite is 114.13 MPa, with only a minor difference. This indicates that the incorporation of the flame-retardant system does not significantly compromise the compressive performance of the material. APP, as a rigid inorganic particle, can partially bear the applied load during compression, providing some compensatory reinforcement. Although the simultaneously introduced NH_2_-MWCNTs possess a high modulus, their relatively low content limits their overall reinforcing effect, making it insufficient to offset the weakening caused by the high APP loading. Figure 10c shows the impact strength of epoxy resin and its composites. Compared with neat epoxy resin, the impact strength of EP/17.5APP/0.1NH_2_-MWCNTs slightly increases to 10.1 MPa. This improvement can be attributed to the incorporation of APP and NH_2_-MWCNTs, which introduce additional interfaces within the epoxy matrix and promote crack deflection and energy dissipation during impact loading. Meanwhile, the NH_2_-MWCNTs enhance interfacial interactions with the epoxy matrix, which may help delay crack propagation. Although the improvement is limited, it still indicates a slight enhancement in the toughness of the composite system. Figure 10d presents the tensile modulus of epoxy resin and its composites. As shown, the tensile modulus increases from 2.3 Gpa to 3.0 Gpa after the addition of APP and NH_2_-MWCNTs. This improvement is mainly attributed to the incorporation of rigid APP particles and high-modulus NH_2_-MWCNTs, which restrict the mobility of epoxy chain segments and increase the stiffness of the composite. In addition, the improved interfacial interaction between NH_2_-MWCNTs and the epoxy matrix facilitates stress transfer at small deformation, leading to an increase in tensile modulus.

### 3.6. Char Residue Analysis

**Figure 11:**
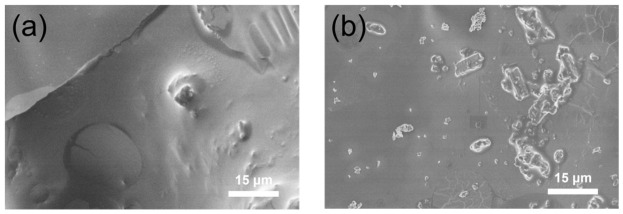
SEM images of the residual carbon of EP and its composites ((**a**) EP; (**b**) EP/17.5APP/0.1NH_2_-MWCNTs).

In order to gain further insight into the flame-retardant mechanism of epoxy resin and its composites, SEM was used to characterize the morphology of the char residues after combustion, as shown in Figure 11. It can be observed that the char layer formed from the neat cured epoxy resin is relatively loose, with numerous pores and cracks. Such a structure provides limited barrier effects during combustion, offering little resistance to heat transfer into the material or the release of combustible gases. In contrast, the char morphology of the EP/17.5APP/0.1NH_2_-MWCNTs composite shows significant improvement. The char layer is flatter and denser, with fewer pores and cracks, exhibiting better continuity and integrity. During combustion, a continuous and compact char layer can serve as an effective barrier to heat and mass transfer, contributing to improved flame-retardant performance of the materials [24].

**Figure 12:**
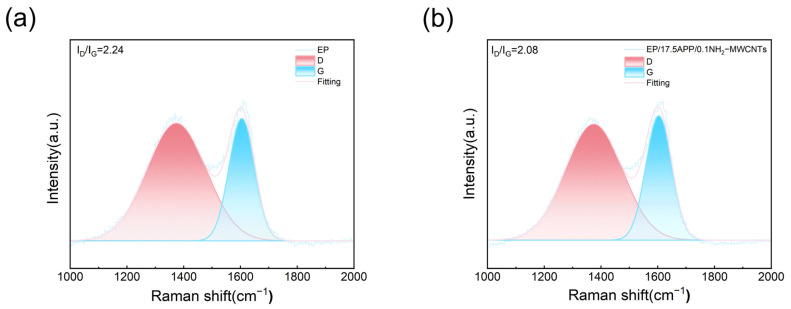
Raman spectra of the residual carbon of EP and its composites ((**a**) EP; (**b**) EP/17.5APP/0.1NH_2_-MWCNTs).

The Raman spectra of epoxy resin and its composites are displayed in Figure 12. Two characteristic peaks located at 1350 cm^−1^ and 1580 cm^−1^ can be clearly identified, corresponding to the D band and G band, respectively. The I_D_/I_G_ ratio is used to characterize the degree of ordering of carbon atoms and to evaluate the graphitization level of the char residues after combustion [25]. Compared with the neat cured epoxy resin, the I_D_/I_G_ ratio of the char residues in the composites containing APP and NH_2_-MWCNTs decreases significantly. Specifically, the I_D_/I_G_ of EP/17.5APP/0.1NH_2_-MWCNTs char decreases from 2.34 for the neat epoxy to 2.08, indicating that APP and NH_2_-MWCNTs can effectively enhance the graphitization degree of the epoxy during combustion. This dense char layer can efficiently block contact between the material and oxygen, thereby improving the flame-retardant performance of the epoxy matrix. These results agree well with the previously observed char morphology and thermogravimetric analysis, further confirming the beneficial effect of the composite flame-retardant system on improving the flame retardancy of epoxy resin.

**Figure 13:**
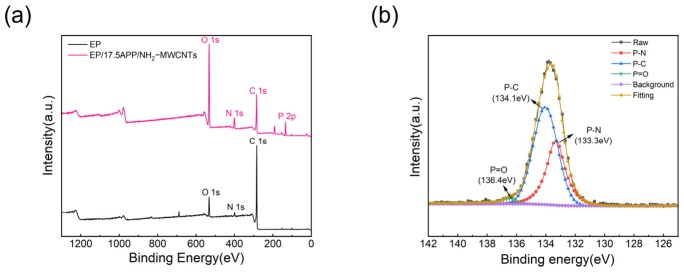
(**a**) XPS spectra of the residual carbon of EP and its composites; (**b**) high-resolution spectra of P 2p.

Figure 13a presents the XPS spectra of the char residues from epoxy resin and its composites. As shown, both char residues contain characteristic C, N, O elements on their surfaces. In the spectrum of the EP/17.5APP/0.1NH_2_-MWCNTs sample, a distinct P 2p peak appears near 133 eV, indicating that the addition of APP successfully introduced phosphorus into the flame-retardant system and participated in the condensed-phase reactions during combustion. High-resolution analysis of the P 2p spectrum is shown in Figure 13b. The peak at 136.4 eV is assigned to P=O structures, confirming that ammonium polyphosphate decomposes at high temperatures to form phosphorus-containing compounds. Peaks observed at 134.3 eV and 133.3 eV correspond to P-C and P-N bonds, respectively, indicating chemical interactions between APP and NH_2_-MWCNTs during combustion. This structure contributes to the formation of a more stable and dense C/P/N composite protective layer on the material surface, effectively enhancing the thermal stability and barrier properties of the char layer, and demonstrating a synergistic flame-retardant mechanism involving phosphorus, carbon and nitrogen.

### 3.7. Flame-Retardant Mechanism Analysis

During the thermal exposure of the composites, APP decomposes to generate polyphosphoric species with dehydrating properties, which promote dehydration and charring of the epoxy resin. Meanwhile, NH_2_-MWCNTs act as a supporting framework in the condensed phase. The amino functional groups on their surfaces can interact with the phosphorus-containing decomposition products, facilitating the formation of a more stable char layer. This char layer effectively blocks heat transfer into the material and reduces the release of combustible gases. In terms of gas-phase flame retardancy, the nonflammable gases such as ammonia and nitrogen released from APP decomposition dilute the concentrations of combustible gases and oxygen in the combustion zone. The incorporation of NH_2_-MWCNTs also enhances the structural strength of the char layer, making it less prone to cracking under high temperatures and allowing it to maintain its barrier function. Additionally, amination improves the dispersion of MWCNTs within the epoxy matrix and strengthens the interfacial interactions, promoting the formation of a more uniform flame-retardant structure during combustion.

## 4. Conclusions

In summary, this study developed a novel flame-retardant system for epoxy resin by incorporating NH_2_-MWCNTs and APP, combining a phosphorus-based flame retardant with inorganic nanomaterials. Structural characterization using FT-IR, XPS, and XRD confirmed the successful modification of COOH-MWCNTs. With the addition of 17.5 wt% APP and 0.1 wt% NH_2_-MWCNTs, the epoxy resin composites exhibited excellent flame-retardant performance, achieving a LOI of 29.6% and a UL-94 vertical burning rating of V-0. In addition, the modified materials exhibited a significantly increased char yield at high temperature, along with improved impact strength and tensile modulus, while the compressive strength remained essentially unchanged. Moreover, the smoke density of the modified materials was notably reduced. The gas-phase and condensed-phase flame-retardant mechanisms of epoxy resin and its composites were investigated by analyzing the char residues.

The phosphorus-containing radicals produced by APP decomposition during combustion capture H· and HO· radicals required for the combustion chain reaction, while NH_2_-MWCNTs can construct a dense, continuous, and highly stable char layer that effectively blocks heat exchange and the release of combustible gases. Through synergistic effects, both components jointly achieve effective flame retardancy for epoxy resin.

## Figures and Tables

**Table 1:** Formulation for Processing of Epoxy Resin and Its Composite Materials.

Sample	EP(wt%)	DDM(wt%)	APP(wt%)	NH_2_-MWCNTs(wt%)
EP	80.0	20.0	0	0
EP/15APP	68.0	17.0	15.0	0
EP/17.5APP	66.0	16.5	17.5	0
EP/0.1COOH-MWCNTs	79.9	19.9	0	0.1
EP/5APP/0.1NH_2_-MWCNTs	75.9	18.9	5.0	0.1
EP/7.5APP/0.1NH_2_-MWCNTs	73.9	18.5	7.5	0.1
EP/10APP/0.1NH_2_-MWCNTs	71.9	18.0	10.0	0.1
EP/12.5APP/0.1NH_2_-MWCNTs	69.9	17.4	12.5	0.1
EP/15APP/0.1NH_2_-MWCNTs	67.9	17.0	15.0	0.1
EP/17.5APP/0.1NH_2_-MWCNTs	65.9	16.5	17.5	0.1
EP/15APP/0.1COOH-MWCNTs	67.9	17.0	15.0	0.1
EP/17.5APP/0.1COOH-MWCNTs	65.9	16.5	17.5	0.1

**Table 2:** Vertical burning ratings and limiting oxygen index test data of EP and its composites.

Sample	t_1_/t_2_ (s)	Rating	Dripping (Yes/No)	LOI (%)
EP	-	No rating	Yes	21.5
EP/15APP	7 s/13 s	V-1	No	27.3
EP/17.5APP	3 s/7 s	V-0	No	28.6
EP/0.1NH_2_-MWCNTs	-	No rating	No	22.0
EP/5APP/0.1NH_2_-MWCNTs	-	No rating	No	22.9
EP/7.5APP/0.1NH_2_-MWCNTs	-	No rating	No	23.7
EP/10APP/0.1NH_2_-MWCNTs	14 s/42 s	V-2	No	25.4
EP/12.5APP/0.1NH_2_-MWCNTs	8 s/19 s	V-1	No	26.9
EP/15APP/0.1NH_2_-MWCNTs	5 s/9 s	V-0	No	28.1
EP/17.5APP/0.1NH_2_-MWCNTs	2 s/2 s	V-0	No	29.6
EP/15APP/0.1COOH-MWCNTs	7 s/11 s	V-1	No	27.7
EP/17.5APP/0.1COOH-MWCNTs	3 s/5 s	V-0	No	29.0

**Table 3:** Thermal analysis data of EP and its composites.

Sample	T_5%_ (°C)	T_max_ (°C)	R_Tmax_ (wt%/min)	C_600_ (wt%)
EP	381.5	395.9	17.5	31.1
EP/17.5APP/0.1NH_2_-MWCNTs	333.4	359.7	12.4	44.2
EP/17.5APP	350.8	373.5	15.7	37.8

## Data Availability

The original contributions presented in this study are included in the article. Further inquiries can be directed to the corresponding authors.
